# An integrative model of new product evaluation: A systematic investigation of perceived novelty and product evaluation in the movie industry

**DOI:** 10.1371/journal.pone.0265193

**Published:** 2022-03-11

**Authors:** Yingyue Luan, Yeun Joon Kim

**Affiliations:** Judge Business School, University of Cambridge, Cambridge, United Kingdom; University of Salento, ITALY

## Abstract

The literature on perceived novelty and product evaluation has diverged into two disparate streams of research. The first stream builds on theories of curiosity and argues that the perceived novelty of a new product benefits product evaluation because it induces curiosity and provides evaluators (e.g., customers) with positive experiences in learning new features of the product and in resolving their curiosity. In contrast, the second stream adopts theories of expectation violations and argues that perceived novelty decreases product evaluation because it violates evaluators’ expectations of a new product and requires burdensome efforts to make sense of the product. The main goal of our research is to resolve this theoretical inconsistency by offering an integrative model of new product evaluation that proposes an inverted U-shaped curvilinear relationship between perceived novelty and product evaluation. Based on this model, we further examine whether a producer’s reputation plays an ironic moderating role in this curvilinear relationship. Utilizing content analysis and big data approaches with a large sample of 49,835 reviews of 147 movies in the movie industry, we found that an evaluator’s perception of the novelty of a new movie benefited product evaluation but only when that perceived novelty was moderate; at higher levels of perceived novelty, the product evaluation decreased. In addition, we compared the curves of high vs. low reputation producers and found that perceived novelty penalized product evaluation of new movies created by high reputation producers.

## Introduction

*Do customers like or dislike new products with high novelty*? This question has been the subject of debate in many professional fields for decades. For example, in the movie industry, some film directors argue that novelty is the key factor for audience satisfaction. As Steven Spielberg once put it, “*The public has an appetite for anything about imagination—anything that is as far away from reality as is creatively possible*.” Other directors have an opposite view. Brad Bird once said, “*Familiarity is all the rage*. *And if you’re doing something that doesn’t have its rhythms preset*, *you know*, *everybody’s a little bit uncomfortable*.” The same debate is ongoing in academic research with disparate theoretical lenses. Some researchers draw on theories of curiosity to suggest that perceived novelty in a new product elicits curiosity, which motivates evaluators to learn more about the product and ultimately gives them a positive and satisfying experience of resolving their curiosity [1, 2]. In contrast, another group of researchers utilizes theories of expectation violations to suggest that novel products generate a negative experience—they violate evaluators’ expectations and bother evaluators to expend extra effort to make sense of the newness of the products [3, 4]. It is important to address this unresolved question because perceived novelty influences the user acceptance [5], financial success [6, 7], and diffusion and adoption of a product [8].

The central purpose of our research is to develop an integrative theoretical model of new product evaluation that can accommodate the two contradictory theories and to provide convincing empirical evidence for this model by utilizing a large volume of customer movie reviews. To study new products and evaluators’ perceptions of these products, an ideal research setting should have high volumes of both supply (i.e., new products) and demand (i.e., customers or evaluators of new products). The movie industry meets these criteria because it has a highly competitive market in which many new products are released every year. This competitive market offers an interesting research opportunity to investigate new products. In addition to the large supply of new products, the movie industry is in high demand as billions of movie tickets are sold every year. More importantly, this industry has established online platforms for customers to evaluate new releases and leave numerous reviews on these platforms.

With a large sample of 49,835 reviews of 147 movies in the U.S., we test our integrative model of new product evaluation, which proposes an inverted U-shaped curvilinear relationship between perceived novelty and product evaluation. This model attempts to integrate the two contradictory perspectives and suggests that a new product should offer evaluators a balanced experience between novelty and familiarity to gain favorable evaluations. Thus, a moderate level of perceived novelty that elicits curiosity but does not violate evaluators’ product expectations would receive the highest level of product evaluations. Research in adjacent areas, such as schema incongruity and product innovation, has hinted at this curvilinear relationship. In line with our model, Calantone, Chan, Cui (2006) [9] proposed the need for a balance between perceived product advantage and familiarity in innovative products. A moderate level of perceived novelty would satisfy such a need and benefit the evaluation and success of innovative products.

Based on this integrative model of new product evaluation, our research further investigates whether a producer’s reputation influences the inverted U-shaped curvilinear relationship between perceived novelty and product evaluation. Past research has shown that evaluators’ perception of a new product and their subsequent evaluations are determined by not only the core characteristics of the new product (e.g., product novelty) but also other peripheral pieces of information, such as a producer’s characteristics [10, 11]. Among many, our research investigates the role of a producer’s reputation because evaluators often seek information about a producer’s reputation and adopt different evaluation standards accordingly [11]. Drawing on past research (see, for example [12, 13]), we expect that producers’ reputation may play an ironic role—although high reputation producers likely earned their reputation by creating novel products in the past, their reputation can become a liability for their future novel products. Specifically, evaluators may appreciate less novelty in new products created by high reputation producers than in those created by low reputation producers. This is because the former (novel) products of high reputation producers may have become archetypes in evaluators’ minds, and evaluators prefer high alignment between these producers’ subsequent new products and their former products.

**Fig 1:**
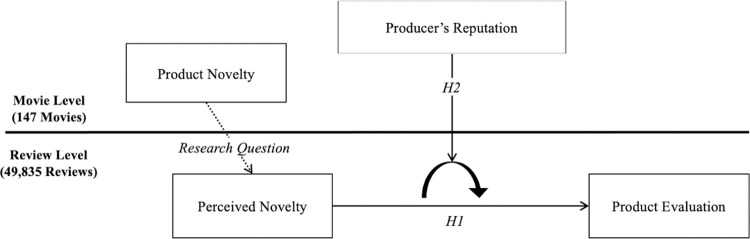
Theoretical model.

Our integrative model of new product evaluation makes notable contributions to the literature. It contributes to the literature on novelty evaluation by resolving the theoretical and empirical inconsistencies in the relationship between perceived novelty and product evaluation. Our study also contributes to the literature on the reverse halo effect by adding a new finding in the context of new product evaluation—producers’ reputation becomes a penalty. Furthermore, our additional analyses offer a surprising finding that speaks to the literature on the producer’s side of novelty, which is conceptually related to, but distinct from, perceived novelty. That is, we found that product novelty was unrelated to the novelty of the product as perceived by customers. This finding advances the research on novelty and offers reasons for the inconsistent results on the relationship between product novelty and the success of new products. Fig 1 depicts our theoretical model.

### Definition of perceived novelty

Perceived novelty refers to the extent to which an evaluator, after deliberately reviewing a new product, acknowledges that the product is different from existing products in a unique and original way. This definition has two key characteristics: subjectivity and deliberate assessment. Perceived novelty accounts for a perceiver’s subjective recognition of a new product’s novelty. This means that perceivers (e.g., customers) perceive and evaluate a new product’s novelty to varying degrees. In addition, our definition accounts for an evaluator’s perception of novelty after he/she thoroughly reviews a new product rather than his/her first impression of it. A first impression can be heavily influenced by how well the product is advertised or framed by the producer (e.g., advertisements of new movies) or by a brief interaction with the product (e.g., watching trailers of new movies), which shapes an individual’s initial evaluation of the product [14]. However, past research has shown that despite the influences of the first impression on the initial evaluation, the subsequent deliberate information processing (e.g., watching the whole movie) tends to override the first impression and update a customer’s initial evaluation [15–18]. Our definition thus considers perceived novelty as an outcome of a person’s deliberate assessments on a new product.

Past research has investigated perceived novelty across various products and ideas in diverse industries (see, our summary in Table 1). For example, Talukdar and Yu (2021) [19] examined customers’ perceived novelty in various virtual reality products. Davis et al. (2017) [20] examined perceived creativity in entrepreneurial pitches and crowdfunding performance. Nevertheless, their theories conceptualize perceived novelty as a perceiver’s *general* psychological responses to newness, which are not unique to a specific product but universally applicable to products regardless of their types and industries. Following past studies, our research engages in the theoretical discussions regarding the general theories of perceived novelty, although we test our theoretical model in the movie industry. Later in the discussion section, we discuss a potential limitation in the generalizability of our empirical setting.

**Table 1:** Summary of perceived novelty research across products, ideas, and industries.

Study	Product/Idea	Industry	Scope of Theorizing Perceived Novelty
Wells, Campbell, Valacich, & Featherman, 2010 [5]	Biometric hand-scanner	Information technology	General
Davis, Hmieleski, Webb, & Coombs, 2017 [20]	Entrepreneurial funding pitches	Crowdfunding	General
S. Lee, Ha, & Widdows, 2011 [21]	High-technology product	Technology	General
Mugge & Dahl, 2013 [4]	Digital camera, washing machine, and hairdryer	Product design	General
Moreau, Markman, & Lehmann, 2001 [22]	Camera	Camera	General
Talukdar & Yu, 2021 [19]	Virtual reality	Virtual reality	General
Weaver, Caldwell, & Sheafer, 2019 [23]	Alternate uses tests and engineering design ideation	Academic, Engineering	General
Zhuang, Toms, & Demartini, 2018 [24]	Internet Browsing	User experience interface	General
Chevalier & Mayzlin, 2006 [6]	Book	Publishing	General
Chen & Xie, 2008 [25]	Movie	Film	General
Nguyen & Hunter, 2021 [26]	Classroom redesign ideas	Education	General

*Note*. For the scope of theorizing perceived novelty, we checked if each paper developed its theory either in a generally applicable way or specific to a certain product, idea, or industry. We found that all papers developed general theories of perceived novelty.

In what follows, we review the literature on perceived novelty and identify two conflicting theoretical perspectives that have produced empirical inconsistencies regarding the relationship between perceived novelty and product evaluation. Then, we present our integrative model of new product evaluation, which incorporates these two contrary perspectives. Based on our integrative model, we further examine how a producer’s reputation may have negative halo effects on the relationship between perceived novelty and product evaluation.

### Perceived novelty and product evaluation of a new product

Past research on the relationship between perceived novelty and product evaluation has diverged into two contradictory perspectives. The first perspective builds on theories of curiosity and suggests that perceived novelty increases product evaluation, as it renders a positive experience of learning and resolving curiosity. Litman and colleagues proposed a model of curiosity that offers a theoretical explanation for why perceived novelty may lead to a favorable evaluation of a new product [1, 2]. They argued that curiosity is aroused by a sense of deprivation—when a person feels that he/she lacks information about a new product (i.e., high perceived novelty), this creates an unsatisfying state (i.e., feeling of deprivation) that triggers an intense desire to know more about the product (i.e., curiosity). This undesirable state is resolved by learning about new features and functions of the new product, which in turn alleviates the person’s need for novel knowledge, reduces the person’s sense of being ignorant, creates positive emotions such as excitement and joy, and ultimately leads to positive attitudes and evaluations towards the new product. However, if a person does not feel that he/she lacks information about the new product (i.e., low perceived novelty), he/she is much less likely to have the positive experience of feeling and resolving curiosity. As a result, he/she may consider the product less interesting or desirable, and ultimately gives a less favorable product evaluation. Past studies in the literature have provided indirect evidence supporting this perspective. For example, Wells et al. (2010) [5] showed that perceived novelty increased the chance of innovation adoption. Davis et al. (2017) [20] found that perceived product creativity was positively related to the crowdfunding performance of a new idea. S. Lee et al. (2011) [21] observed that perceived uniqueness led to positive emotional arousal towards a new product.

In contrast, the second perspective focuses on the uncertainty and risk associated with novelty and suggests that perceived novelty may lower product evaluation. Researchers advocating this perspective have argued that people prefer to maintain the status quo and use familiar products because doing so provides a sense of comfort and security, whereas the use of novel products is perceived as disturbing and uncertain [4, 27]. Mueller et al. (2012) [3], for instance, showed that people held a negative bias towards novelty because novelty involved ambiguity and risk. Perceived uncertainty and risk about a new product are aversive experiences that elicit negative emotions, which reduce people’s intention to adopt the new product [28]. Marketing research on product categorization reaches the same conclusion drawing on theories of expectation violations. Marketing researchers have suggested that customers tend to make sense of a new product using product categories they are already aware of [29, 30]. If the new product cannot be categorized within existing product categories, people feel confused and uncomfortable [31]. This is because to understand the new product, customers need to devote extra cognitive effort to generate a new product category or rearrange the whole system of product categories in their mind [32]. Mugge and Dahl (2013) [4] provided indirect evidence supporting this claim by showing that less novel product designs reduced the learning cost of a new product and thus improved the customer evaluation of that product. Moreover, Moreau, Markman, et al. (2001) [22] found that a novel product that failed to fit within customers’ expected categorization resulted in less favorable attitudes towards its performance, which in turn lowered their product evaluation.

The main goal of our research is not to dispute the two perspectives in the extant literature but rather to offer a broader theoretical model that incorporates the two simultaneously and to resolve the empirical inconsistencies of perceived novelty and product evaluation. In line with the first perspective, we suggest that the novelty perceived by evaluators (e.g., customers) can be desirable, as it elicits curiosity and offers a chance to experience the pleasant feeling of appeasing curiosity. For example, Christopher Nolan, director of The Dark Knight trilogy and Inception, once said, “One of the really important things in Hollywood culture is an absolute acknowledgement that freshness and novelty are key”. At the same time, we acknowledge the possibility that perceived novelty provokes discomfort and requires extra cognitive effort from customers to make sense of a new product. Walt Disney once said, “Do what you do so well that they will want to see it again and bring their friends.” Our research attempts to integrate these two opposing perspectives by answering the new question of whether it may be too much perceived novelty that makes evaluators uncomfortable, while a moderate level of perceived novelty leads to the most favorable evaluations.

Although the possibility of a curvilinear relationship between perceived novelty and product evaluation has not yet been examined, it has been proposed or implied by some extant theories. For example, theories of schema incongruity suggest that products that are viewed as moderately incongruent with an evaluator’s expectations are preferred to both completely congruent and extremely incongruent alternatives [33]. Even though people prefer predictability over unpredictability, incongruity that can be resolved relatively easily (i.e., moderate incongruity) is deemed rewarding and elicits positive affects [34]. Furthermore, in a more recent study, Calantone et al. (2006) [9] found a tension between the perceived advantage and familiarity of innovative products: On the one hand, customers perceived innovative products as useful and advantageous (e.g., creating a positive experience of eliciting and resolving curiosity); on the other hand, customers felt discomfort due to the unfamiliarity of the innovative products. Based on these findings, the authors proposed the possibility of a curvilinear relation between innovativeness ratings and product evaluation. That is, a moderate level of innovativeness could be an optimal point that creates harmony between perceived product advantage and familiarity. Min and Schwarz (2021) [35] also emphasized that novelty offered unknown opportunities as well as unknown risks, requiring a balanced consideration. A too-low level of perceived novelty that leads to familiarity but not to product advantage may make a new product seem mediocre and dull, resulting in an unsatisfactory product evaluation. A too-high level of novelty that signals product advantage but not familiarity may make the product seem incomprehensible and thus lead to a less favorable product evaluation. However, a moderate level of perceived novelty can communicate both product advantage and familiarity and may elicit the most favorable evaluations. Drawing on these theories, we propose the following hypothesis:

*Hypothesis 1: Perceived novelty of a new product has an inverted U-shaped curvilinear relationship with an evaluator’s product evaluation such that the perceived novelty is the most beneficial to product evaluation when it is at a moderate level*.

### The role of a producer’s reputation

When evaluating a new product, evaluators also consider the characteristics of its producer for reference [11]. A producer refers to a person, or an entity, who represents the identity of all production parties. In the movie industry, multiple parties may represent the role of a producer (e.g., the chief director of a film, production companies, screenwriters). Our research focuses on chief directors because (1) compared to other production parties, the audience pays greater attention to and relies on the reputations of chief directors in deciding whether to watch new movies, and (2) the audience tends to perceive that chief directors represent all production staff involved in a movie [36]. Also, it is important to note that in the movie industry, there is a specific role of the “producer” who oversees film production. The use of the term “producer” in our research does *not* refer to this specific role but refers to its general meaning—a person who represents the identity of all production parties [11].

A new product usually involves a certain level of ambiguity regarding its quality and functionality [11], and thus, evaluators often look for cues from the producers to derive information about its quality and make use of these cues to perform heuristic evaluations of the new product [10]. Researchers have suggested that a producer’s reputation is one of the cues that can be easily obtained by evaluators and attract them to purchase and use a new product [37]. For example, imagine that a new movie directed by Steven Spielberg is just released. Many people will not hesitate to watch the movie (even without knowing its topic, genres, or contents) because he is one of the most renowned directors. However, being attracted to the movie does not mean that people will favorably evaluate the movie after watching it. In fact, people often criticize movies directed by reputable directors, and thus, it is not unusual that the movies of many reputable directors fail to become box office hits. Even some of Spielberg’s movies have received unsatisfactory evaluations from audiences. In other words, although a producer’s reputation does offer cues regarding the product and sets people’s expectations for it, that reputation alone is less likely to directly influence evaluators’ product evaluation. Rather, it may interact with a core characteristic of the new product, such as novelty, to shape product evaluation. Based on this conclusion, our research questions whether people’s product evaluations are a function of the director’s reputation and perceived novelty of a new movie—e.g., *in regard to the novelty of a movie produced by Spielberg*, *would people want to see a typical Spielberg style movie or a very novel style movie that differs markedly from other movies*, *including Spielberg’s past movies*? *Do people expect a different level of novelty if the movie is created by a less famous director*?.

Our research answers this interesting yet unexplored question by suggesting that evaluators may appreciate more novelty in a new product created by a low reputation producer than in one created by a high reputation producer. A producer develops an identity by creating a portfolio of past representative works [38, 39]. The typical process of establishing a recognizable identity is as follows: (1) in their early career, producers imbue their lines of products with novel patterns, or product archetypes, (2) the product archetypes are recognized as novel both by laypeople and by people in their field (i.e., experts and colleagues in the subject area), and (3) the repeated public recognition (e.g., awards and prizes) of the producers’ works leads to their product archetypes becoming representative of their reputation. In other words, the fact that a producer has a high reputation means that a product archetype can generally be found among the producer’s past works and that people expect the producer’s subsequent products to be highly in line with his/her established identity and appreciate when they are [40]. Thus, evaluators are more likely to favorably evaluate the subsequent products of a high reputation producer when those products fit well with the producer’s identity and to penalize the products when they are perceived to differ greatly from the producer’s identity. In contrast, for a less reputable producer who does not have product archetypes established in people’s minds, evaluators are more likely to emphasize how unique his/her product is compared to existing products. Supporting this conclusion, researchers in the literature on brand extension found that when consumers perceived *commonalities* between a new product and existing products of an established brand (i.e., producer), they felt pleased and formed favorable attitudes towards the new product because they could easily project the positive qualities of existing products onto the new product. However, such favorable attitudes resulting from perceived commonalities were not observed among less established brands [41–43]. Thus, we propose the following hypothesis:

*Hypothesis 2: A producer’s reputation moderates the curvilinear relationship between perceived novelty and evaluation of a product such that the peak point of the inverted U-shaped curve comes earlier, and product evaluation drops more drastically after the peak point when the producer has a higher reputation*.

### Research question: The relationship between product novelty and perceived novelty

In the literature, another concept related to perceived novelty is product novelty, which refers to the degree to which a new product creates a novel product category or integrates extant product categories in novel ways [12, 44–48]. There are two assumptions underlying this definition–(1) a novel product creates a completely new product category because the current category cannot categorize such a novel product, or (2) a novel product combines extant product categories in a unique way. Past researchers [12, 44–48] conceptualized and operationalized product novelty relying on the second assumption (i.e., novel combinations of extant product categories) because in reality, it is extremely rare for novelty to be so radical that a new product creates a completely new product category. For example, researchers have measured product novelty by considering whether a new financial report uses novel combinations of extant *repertoires* (or, in their words, portfolios of framing [46]) and how much researchers use *novel* c*ombinations of upper-level keywords* in research proposals to describe their new work (see, for example [44, 45, 47]).

Product novelty may or may not predict customers’ perceived novelty of a new product because people do not always perceive the same stimuli in the same way. As a result, customers’ novelty perception may not align with the product novelty intended by the producer [31, 49, 50]. Nevertheless, researchers, who examined product novelty, have assumed that product novelty always shapes customers’ perceived novelty–i.e., product novelty invariably makes customers perceive the new product as novel. Yet to our knowledge, this assumption has not been tested. In fact, some past theories challenge this assumption. For example, Rindova and Petkova (2007) [31] argued that a product’s characteristics may not be accurately perceived by customers in the intended way due to the expectation gap between the producer and evaluators. Adner and Levinthal (2008) [49] also showed that novel activities were perceived differently by actors and audience members—stakeholders often failed to recognize entrepreneurs’ genuine intentions about their novel activities (i.e., entrepreneurship). Finally, Fuchs and colleagues (2019) [50] found that products were often overvalued by producers, which led to negative evaluations and rejections from customers. In sum, these studies question the validity of the assumed link between product novelty and a customer’s perceived novelty, which might have contributed to previous papers’ empirical inconsistencies regarding the relationship between product novelty and the success of a new product (see, for example [44, 45]).

Our research asks an open-ended research question regarding the relationship between product novelty and perceived novelty. The reason why we set an open-ended question is that as we showed above, there are theories offering opposite predictions regarding this relationship. As mentioned above, the theoretical accounts of the relationship between product novelty and the success of a new product are inconsistent—the research on product novelty has frequently assumed this relationship to be positive, but theories in other research have argued that there may be no relationship. Answering this question can be beneficial in two ways. First, by examining the strength of this relationship, we can empirically show that product novelty and perceived novelty are distinct concepts that should be examined independently. Second, we can resolve the inconsistent theoretical accounts regarding the relationship between product novelty and perceived novelty.

*Research Question: Is product novelty positively related to a customer’s perceived novelty of a new product?*

## Materials and methods

### Procedure

We collected data from IMDb, an online database for movies, television programs, and other content streaming online. We chose IMDb because it is one of the largest and most authoritative platforms for information related to movies, and it offers a feature that allows users to leave ratings and reviews. An increasing number of studies have begun exploring the richness and diversity of IMDb data (see, for example [51–53]). We cross-check the data collected from IMDb with other sources, such as The Movie Database (TMDb) and Box Office Mojo (boxofficemojo.com).

The data collection focused on movies released in the U.S. in 2016. For movies with multiple release dates, we used the first date that was neither a premiere date nor a date for showing in select theaters, as this date is generally the actual movie release date. We obtained information on features of each movie from different webpages in IMDb: the genres and languages were found on the movie’s main webpage, the directors and actors/actresses were found on the full cast and crew page, and the awards and nominations were found on the awards page. We retrieved information on the awards of directors and actors/actresses from their award pages. We collected movie reviews from the respective review pages in IMDb. Each review includes a rating for the movie, a review title, the date of the review, and the review text. We excluded movies with fewer than 100 reviews to ensure the representativeness and validity of the movie reviews. After data collection, we conducted data cleaning, especially text cleaning on the review dataset. We removed all emojis, URLs, English stop words, and punctuations. We then excluded reviews that were not written in English, reviews with fewer than ten words, and reviews without ratings. As a result, the final dataset included 49,835 reviews for 147 movies. On average, each movie had 339.01 reviews (*SD* = 323.56) and 129.10 words (*SD* = 108.17). All data processing and variable computations were conducted using R (version 4.0.2).

### Dependent variable

#### Product evaluation

We used each reviewer’s rating from his/her movie review. These ratings are given on a ten-point Likert scale from one, representing a poor product evaluation, to ten, representing an excellent product evaluation. The average rating was 6.34 (*SD* = 2.88).

### Independent variables

#### Perceived novelty

To capture the degree of perceived novelty of the movie in each review, we adopted a content analysis approach. Following the method of Uotila et al. (2009) [54], we quantified the perceived novelty of the movie in each review with a two-step procedure: first, we created a lexicon; second, we calculated the relative number of novelty-related words compared to the total number of words in each review. To build the lexicon, we started with existing word lists developed by past researchers [54, 55]. These word lists were intended to quantify the level of explorative and exploitative orientations. We adapted the word list for explorative orientation and modified it based on our rigorous reviews of the relevant literature (e.g., the novelty and creativity literature) and a subset of movie reviews. Most importantly, during this process, we created a lexicon conforming to the conceptual definition of perceived novelty that we elaborated in the theory section. As a result, we came up with a list of 21 word stems, and all possible forms of the word stems were used in our calculation of the relative amount of novelty. We used the following word list: novel*, *different*, *unusual*, *new*, inspir*, unique*, radical*, twist*, edge*, variation*, fresh*, origina*, strange*, unfamiliar*, revolution*, experiment*, discover*, *odd*.*, *avant-garde*, ingenious*, and groundbreaking*. When we extracted the words from review texts, we ensured that there were no negation words prior to the focal word in the list. To quantify the relative amount of perceived novelty in a review, we calculated the proportion of novelty words by the following formula:

$$Perceived\;novelty=\frac{the\;number\;of\;novelty\;words\;in\;a\;review}{the\;total\;number\;of\;words\;in\;a\;review}$$ (1)

#### Producer’s reputation

We measured the producer’s reputation by using each director’s past award information, i.e., the number of awards he/she had won before the current movie was released. The data were collected from each director’s IMDb award page, which contains information on past award records. We first collected all award information and then counted how many awards each director had won prior to and during 2016. For movies with multiple directors, we used the average number of awards across the directors. The average number of director awards was 15.03 (*SD* = 27.40).

#### Product novelty

We also measured product novelty to answer our research question and to control it in our analyses. Following past researchers (see, for example [46]), we calculated it using higher-order categories for classifying or framing movies. Specifically, we used *genres* as the categories for computing product novelty. Genres play an important role in categorizing motives and providing templates for customers to understand a specific movie, and filmmakers thus frequently use genres to advertise their movies. Austin (1989, p. 75) [56], for example, stated that people “have film type preferences and can articulate their preferences, frequently by employing commonly used genre labels.” That is, information about a movie’s genres offers an overarching framework that facilitates communication about the movie to the audience as well as the audience’s understanding of the movie [57].

In line with the definition of product novelty, our operationalization captures whether current, popular combinations of genres can categorize a new product. If extant combinations of genres can readily categorize the new product, our operationalization lowers its novelty; if the new product requires a new combination of genres, our operationalization increases its novelty. There are 20 unique genres used by IMDb: comedy, drama, romance, action, history, thriller, war, adventure, fantasy, science fiction, animation, family, horror, mystery, crime, music, biography, western, musical, and sport. We calculated the product novelty score for each movie as follows: (1) We computed the percentages of each combination of genres for movies released during the five years prior to 2016. The data related to movies from 2011–2015 were collected separately to compute product novelty. (2) For each movie in the 2016 dataset, if the genre combination for the movie had appeared before, the product novelty score was calculated with the formula “1 –the percentage of its genre combination in the five-year dataset.” For example, assume that movie ‘A’ released in 2016 was featured as a specific combination of *adventure* and *comedy*, and the percentage of movies with this genre combination in the past five years (from 2011 to 2015) was 0.8. Then, the product novelty score for movie ‘A’ was 0.2 (calculated by “1–0.8”). If a movie had a genre combination that was not previously developed, the product novelty of this movie was 1 (calculated by “1–0”). There were 705 movies released in the U.S. during 2011 and 2015, and among them, there were 274 unique genre combinations. We then conducted min-max normalization and transformed the values into a decimal between 0 and 1. The average product novelty was 0.71 (*SD =* .34).

### Control variables

In all analyses, we controlled for several movie-level variables: the number of reviews, the reputation of the lead actors, and the director’s age. The number of reviews is the total number of reviews each movie had. We collected the award information of the lead actors in each movie in their credit order and computed their reputation following the same approach that we used for the producer’s reputation. We also calculated the age of directors as of 2016 and used the average director age if a movie had multiple directors. We used the average age of all directors in our dataset for directors whose age was not publicly available. We did not include dummy variables for individual genres again since we considered the combinations of genres in examining product novelty.

## Results

Means, standard deviations, and correlations for all variables are presented in Table 2. To test our hypotheses and research question, we conducted hierarchical linear modeling (HLM) using R to address the nested nature of the data and simultaneously capture the effects of variables at different levels [58]. We used two-level models: there were 49,835 reviews at level 1, and these reviews were nested in 147 movies at level 2. The score for perceived novelty (i.e., the linear term of perceived novelty) was entered as a predictor at level 1. To investigate the curvilinear relationship between perceived novelty and product evaluation, we also added the quadratic term of perceived novelty at level 1. To examine the moderating effect of a producer’s reputation, we tested the cross-level interaction between perceived novelty (level 1) and a producer’s reputation (level 2). We group-mean centered level-1 predictors to minimize any potential problem with multicollinearity and to better interpret the results [59, 60], and we grand-mean centered level-2 predictors to improve the computation and interpretation of cross-level interactions [61]. To test the appropriateness of HLM, we estimated null models to check the between-movie variability of the intercept (τ_00_) and the intraclass correlation coefficient (*ICC*) for the dependent variable. For product evaluation, we found significant between-movie variance (τ_00_ = 1.07, *p* < .001, *ICC*(1) = .13; Table 3, Model 1). This result exhibited the nested nature of our data and showed that it was appropriate to adopt multilevel modeling.

**Table 2:** Means, standard deviations, and correlations of variables.

Variables	M	SD	1	2	3	4
Level 1: review level						
1. Product evaluation	6.34	2.88				
2. Total words	129.10	108.17	.07***			
3. Perceived novelty	.01	.01	.09***	-.05***		
Level 2: movie level						
1. Director’s awards	15.03	27.40				
2. Number of reviews	339.01	323.56	.06			
3. Director’s age	48.57	9.78	.37***	.03		
4. Stars’ awards	17.55	14.70	.15	.24**	.22**	
5. Product novelty	.71	.34	-.02	.03	-.09	-.07

*Note*. *N* movies *=* 147, *N* reviews = 49,835; *p < .05.

**p < .01.

***p < .001. All tests 2-tailed.

**Table 3:** HLM results on product evaluation.

Variables	Product evaluation				
	(1)	(2)	(3)	(4)	(5)
Level 1 variables					
Intercept	6.24*** (.09)	6.29*** (.09)	5.66*** (.44)	5.86*** (.47)	5.87*** (.47)
Perceived novelty		40.45*** (2.72)	39.83*** (2.73)	39.87*** (2.73)	40.94*** (2.65)
Perceived novelty^2^		-420.40*** (46.49)	-406.42*** (46.60)	-407.49*** (46.75)	-446.09*** (46.80)
Level 2 variables					
Number of reviews			.00* (.00)	.00* (.00)	.00*** (.00)
Stars’ awards			.00 (.01)	0.00 (.01)	0.00 (.01)
Director’s age			.01 (.01)	.01 (.01)	.01 (.01)
Product novelty			.33 (.24)	.32 (.24)	.31 (.24)
Director’s awards				.00 (.00)	.00 (.00)
Cross-level interaction					
Perceived novelty × Director’s awards					.35*** (0.11)
Perceived novelty^2^ × Director’s awards					-9.20*** (2.33)
Variance component					
Residual σ^2^	7.26	7.11	7.10	7.10	7.10
Intercept τ_00_	1.07	1.04	1.03	1.05	1.05
Slope variance τ_11_		557.36	559.10	562.95	498.63
Slope variance τ_21_		108918.04	109893.60	111928.10	97166.10
Slope variance τ_11_ explained by director’s awards ^a^					.11
Slope variance τ_21_ explained by director’s awards ^b^					.13

*Note*. *N* movies *=* 147, *N* reviews = 49,835.

*p < .05.

**p < .01.

***p < .001. All tests 2-tailed. Standard errors in parentheses.

^a^ $R_{Level\;2}for\;slope\;\tau_{11}=\frac{\tau_{11}of\;model\;4-\tau_{11}of\;model\;5}{\tau_{11}of\;model\;4}$.

^b^ $R_{Level\;2}for\;slope\;\tau_{21}=\frac{\tau_{21}of\;model\;4-\tau_{21}of\;model\;5}{\tau_{21}of\;model\;4}$.

### Test of hypotheses

**Fig 2:**
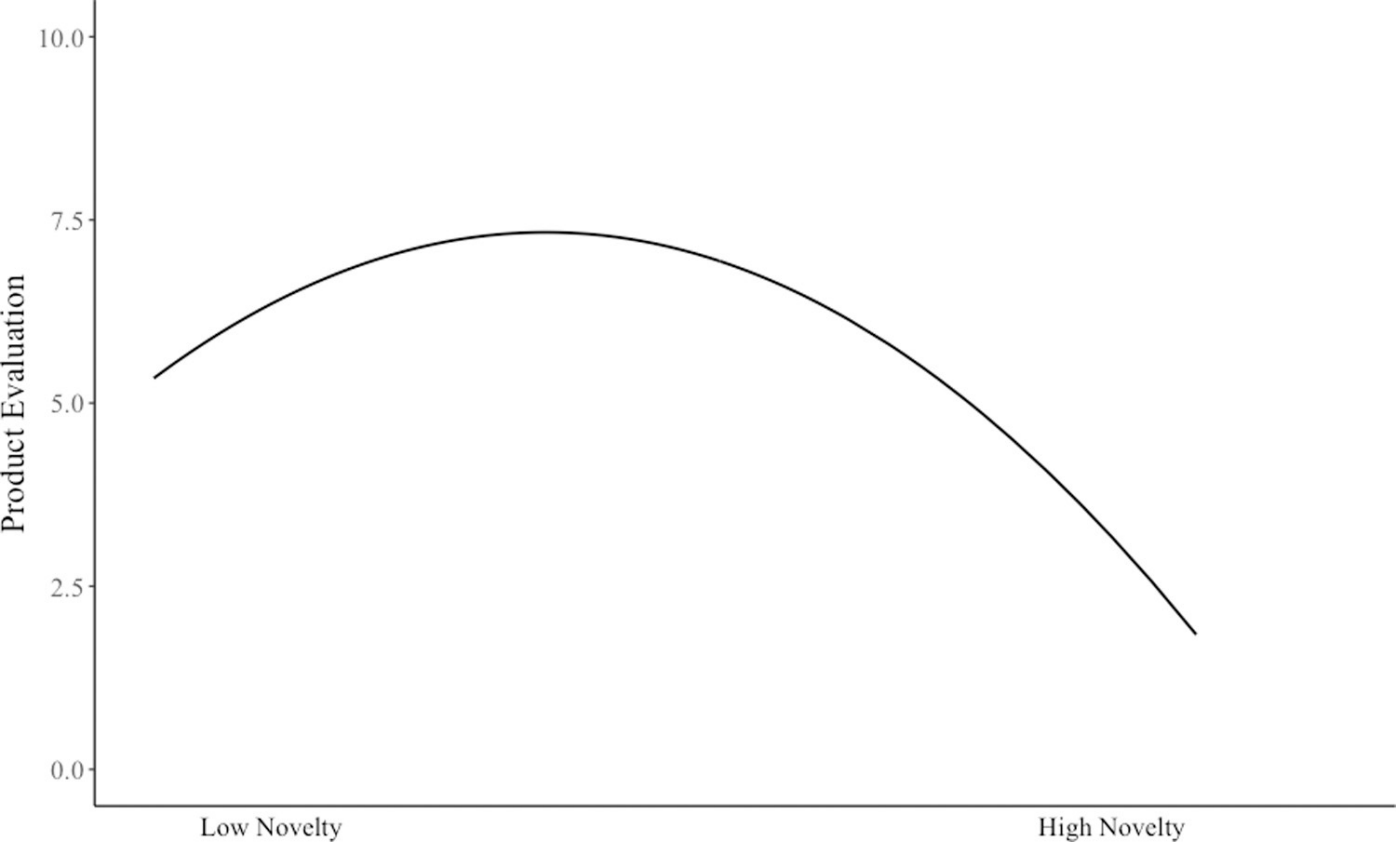
Curvilinear relationship between perceived novelty and product evaluation.

Hypothesis 1 proposed that perceived novelty of a new product has an inverted U-shaped relationship with an evaluator’s product evaluation such that product evaluation is the highest at a moderate level of perceived novelty. To test inverted U-shaped curvilinearity, researchers have suggested checking whether a significantly positive linear term and a significantly negative quadratic term are found simultaneously [62]. The results (Table 3, Model 3) showed that the coefficient for perceived novelty was significantly positive (γ = 39.83, *p* < .001) and that the quadratic term of perceived novelty was significantly negative (γ = -406.42, *p <* .001). To further understand this curvilinear relationship, we depicted the curve in Fig 2, which shows that the pattern of the curve was consistent with the expected inverted U-shape. Thus, Hypothesis 1 was supported.

Hypothesis 2 predicted that a producer’s reputation moderates the curvilinear relationship between perceived novelty and product evaluation. The results (Model 5 in Table 3) showed that the moderating effect of a producer’s reputation on the curvilinear relationship between perceived novelty and product evaluation was significant (the interaction of perceived novelty and the producer’s reputation: γ = .35, *p* < .001; the interaction of the quadratic term of perceived novelty and the producer’s reputation: γ = -9.20, *p* < .001). The cross-level interaction accounted for 11% of the slope variance in the relation between perceived novelty and product evaluation and 13% of that in the relation between the quadratic term of perceived novelty and product evaluation.

Next, we conducted three additional analyses to examine various characteristics of this moderation. First, we conducted simple slope tests to evaluate whether the relationship (slope) between the independent variable (the quadratic term of perceived novelty) and dependent variable (product evaluation) was significant at the first and third quantiles of our moderator (i.e., producer’s reputation) [62–65]. Simple slope tests showed that the effect of the quadratic term of perceived novelty on product evaluation was significant both when the level of a producer’s reputation was high (*b* = -476.40, *p* < .001) and when it was low (*b* = -310.90, *p* < .001). These results showed that the curves for both high and low producer’s reputations were statistically significant.

Second, we conducted a slope difference test to evaluate whether the strengths of the two curves differed significantly. The results showed that the curvilinear relationship between perceived novelty and product evaluation for a high producer’s reputation was stronger than that for a low producer’s reputation (*z* = -14.51, p < .001). This means that the curve of high reputation producers was steeper than that of low reputation producers—i.e., after the peak point, greater perceived novelty was more harmful for high reputation producers than for low reputation producers.

**Fig 3:**
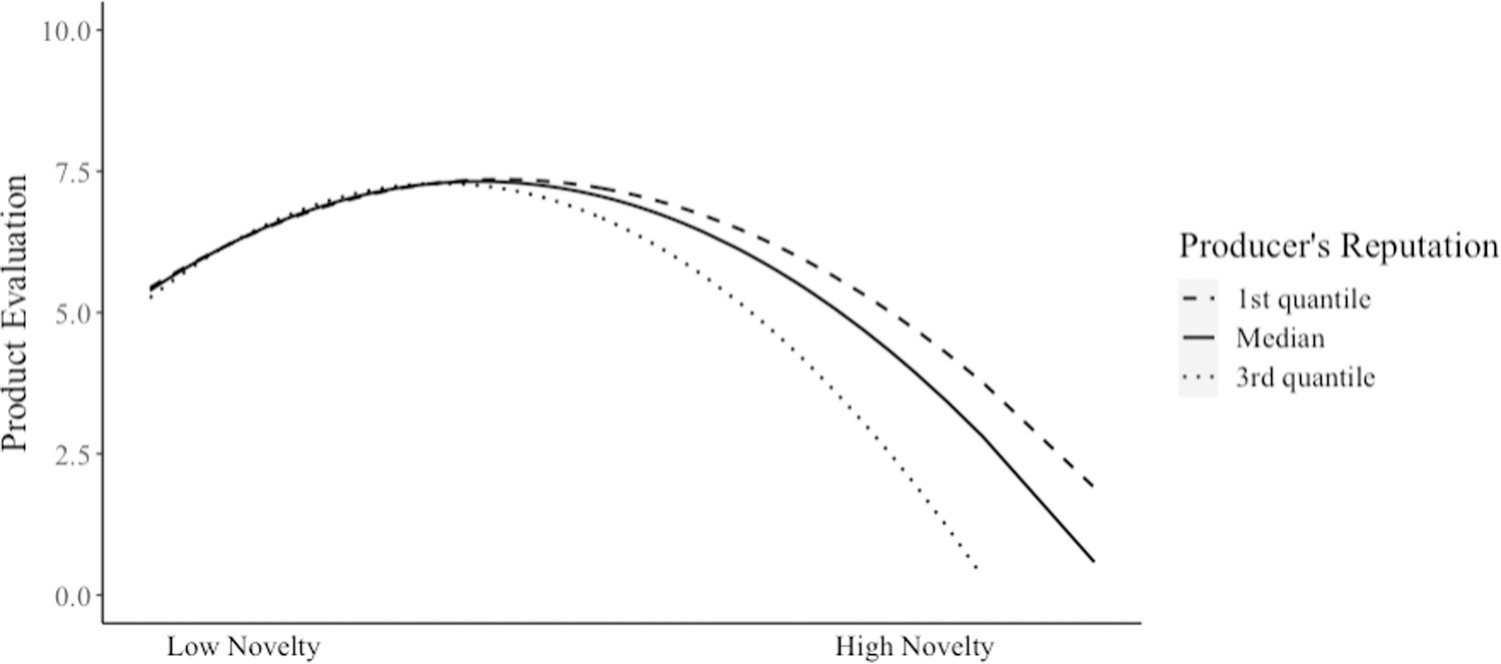
Moderating effects of a producer’s reputation on perceived novelty and product evaluation.

Third, we calculated the specific locations of the peak points for both high and low producer’s reputations. The optimal level of perceived novelty led to the highest product evaluation for low reputation producers’ products (the group-mean centered perceived novelty score at a peak equal to .06; product evaluation score at a peak equal to 7.36) and was one and a half times higher than that for high reputation producers’ products (perceived novelty score (group-mean centered) at a peak equal to .04; product evaluation score at a peak equal to 7.28). This result showed that the peak point of the curve of low reputation producers appeared later than that of high reputation producers. In sum, our results indicated that the novel movies created by producers with higher reputations were penalized by evaluators because for these movies, evaluators appreciated less novelty (i.e., earlier peak point) and rapidly lowered their ratings when they found more novelty after the peak point. Thus, Hypothesis 2 was supported (Fig 3).

Finally, we tested our research question regarding the nature of the relationship between product novelty and perceived novelty. We conducted a cross-level analysis using HLM because product novelty was calculated at the movie level and perceived novelty occurs at the review level. The results showed that product novelty was *not* significantly related to perceived novelty (γ = .00, *p =* .35). This finding supports the claim that perceivers (or evaluators) can fail to recognize the novelty intended by producers (see, for example [31, 49]). More importantly, it shows that product novelty and perceived novelty are two distinct, even unrelated, concepts that should be investigated separately in the literature.

### Supplementary analyses

Another important aspect of new product evaluation is the amount of effort reviewers spend to convey their opinions and thoughts. Research has shown that the length of a review, as a measure of the amount of effort expended by the evaluator in making his/her review, is positively related to how helpful customers find the review [66, 67]. People generally find longer reviews more helpful because these reviews contain more elaborate information and have a higher likelihood of offering critical information that can address audience members’ needs and curiosity [68]. In line with our hypotheses for product evaluation, it is possible that evaluators exert the greatest effort when they find a moderate level of novelty in movies. This is because, as we found above, evaluators appreciate a moderate level of perceived novelty in movies, and they are thus likely to believe that describing and sharing their thoughts on such movies on the IMDb platform is worth the effort. Thus, in additional analyses, we also tested the curvilinear relationship between perceived novelty and evaluators’ effort in their reviews. We operationalized an evaluator’s effort by calculating the total number of words in his/her review.

In this analysis, we used generalized linear mixed modeling with negative binomial in R because the total number of words was a count variable. We found an overdispersion of the data (dispersion ratio >1, *p* < .001); thus, negative binomial analysis was selected over Poisson analysis [69, 70]. To examine the appropriateness of multilevel negative binomial analysis, we again examined the between-movie variability of the intercept (τ_00_) and ICC for the dependent variable. We found significant between-movie variance in the evaluator’s effort variable (τ_00_ = .03, *p* < .001, ICC(1) = .02; Table 4, Model 1).

**Table 4:** GLMM with negative binomial results on efforts in evaluation.

Variables	Effort in evaluation				
	(1)	(2)	(3)	(4)	(5)
Level 1 variables					
Intercept	4.83*** (.01)	4.90*** (.01)	4.85*** (.07)	4.93*** (.07)	4.93*** (.07)
Perceived novelty		21.71*** (1.61)	21.59*** (1.61)	21.61*** (1.61)	21.74*** (1.58)
Perceived novelty^2^		-1102.40*** (70.40)	-1100.38*** (70.26)	-1100.89*** (70.32)	-1103.83*** (69.60)
Level 2 variables					
Number of reviews			.00 (.00)	.00 (.00)	.00 (.00)
Stars’ awards			.00** (.00)	.00* (.00)	.00* (.00)
Director’s age			.00 (.00)	-.00 (.00)	.00 (.00)
Product novelty			.05 (.04)	.05 (.04)	.05 (.04)
Director’s awards				.00** (.00)	.00*** (.00)
Cross-level interaction					
Perceived novelty × Director’s awards					0.13* (0.06)
Perceived novelty^2^ × Director’s awards					-3.81 (2.46)
Variance component					
Residual σ^2^	3.24	3.80	3.81	3.81	1.95
Intercept τ_00_	.03	.03	.02	.02	.02
Slope variance τ_11_		290.02	289.65	290.70	274.60
Slope variance τ_21_		574412.41	573009.64	574329.04	557280.17
Slope variance τ_11_ explained by director’s awards ^a^					.06
Slope variance τ_21_ explained by director’s awards ^b^					.03

*Note*. *N* movies *=* 147, *N* reviews = 49,835.

*p < .05.

**p < .01.

***p < .001. All tests 2-tailed. Standard errors in parentheses.

^a^ $R_{Level\;2}for\;slope\;\tau_{11}=\frac{\tau_{11}of\;model\;4-\tau_{11}of\;model\;5}{\tau_{11}of\;model\;4}$.

^b^ $R_{Level\;2}for\;slope\;\tau_{21}=\frac{\tau_{21}of\;model\;4-\tau_{21}of\;model\;5}{\tau_{21}of\;model\;4}$.

**Fig 4:**
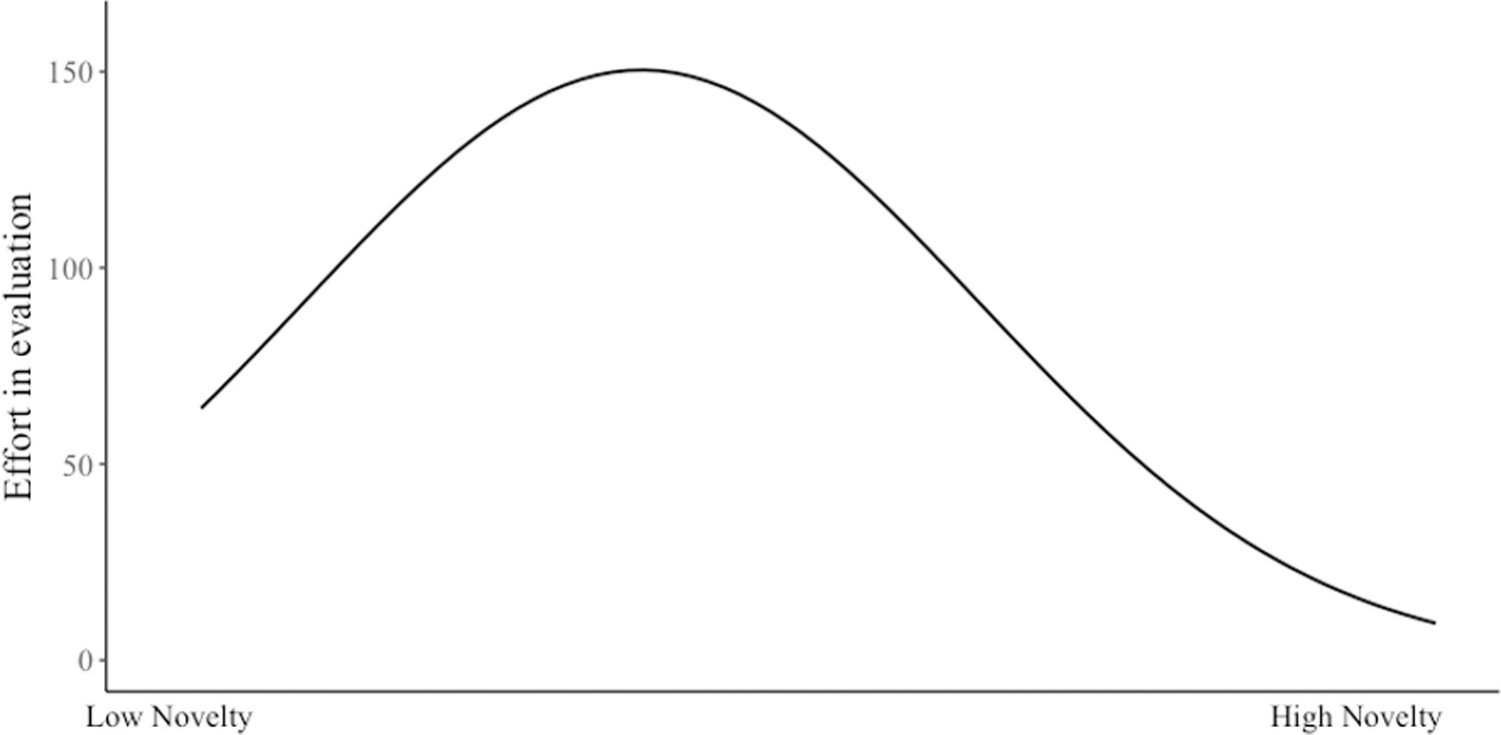
Curvilinear relationship between perceived novelty and efforts in evaluation.

The results showed a significant curvilinear relationship between perceived novelty of a new movie and an evaluator’s effort. As shown in Table 4 (Model 3), the coefficient for the linear term of perceived novelty was significantly positive (γ = 21.59, *p* < .001), and the quadratic term of perceived novelty was significantly negative (γ = -1100.38, *p <* .001). These results proved the inverted U-shaped curvilinearity. Fig 4 depicts the curvilinear relationship. Finally, we tested the moderating effect of a producer’s reputation on the relationship between perceived novelty and an evaluator’s effort and found a nonsignificant result (Table 4, Model 5)—the moderating effect of a producer’s reputation on the relationship between perceived novelty and an evaluator’s effort was significant only at the linear term (the interaction of perceived novelty and a producer’s reputation: γ = .13, *p* < .05) but not at the quadratic term (the interaction of the quadratic term of perceived novelty and a producer’s reputation: γ = -3.81, *p* = .12).

## Discussion

The main goal of our research was to propose an integrative model of new product evaluation to resolve theoretical and empirical inconsistencies regarding the relationship between perceived novelty and product evaluation. With a sample of 49,835 reviews for 147 movies, we found an inverted U-shaped relationship between perceived novelty and product evaluation, suggesting that a moderate level of perceived novelty in a movie leads to the highest product evaluation of that movie. In addition, we found that this relationship was moderated by the producer’s reputation. The results showed that evaluators were more likely to penalize novel movies produced by high reputation directors than novel movies produced by low reputation directors. Specifically, we found that the peak point in the curve for the movies of high reputation producers came earlier than that for the movies of low reputation producers, meaning that evaluators appreciated novelty from new movies produced by high reputation producers less than novelty from new movies produced by low reputation producers. Furthermore, when perceived novelty exceeded its peak point in the curve, product evaluations of high reputation producers’ movies dropped in a steeper manner than those of low reputation producers’ movies. That is, evaluators more harshly penalized novel movies by high reputation producers than novel movies by low reputation producers. In addition, the analyses testing our open-ended research question showed a surprising result—product novelty was unrelated to perceived novelty. This result supports the views that customers vary in terms of their perceptions of a product’s characteristics [31, 49, 50].

### Theoretical and managerial implications

Our research provides several theoretical contributions to the literature. First, we offer an integrative model for new product evaluation that incorporates two conflicting theoretical perspectives on the relationship between perceived novelty and product evaluation. The first perspective builds on theories of curiosity and suggests that the higher perceived novelty of a new product is, the higher its product evaluation. This is because perceived novelty elicits curiosity and provides an opportunity to resolve this curiosity as evaluators learn more about the new product [1, 2]. In contrast, another perspective takes the theoretical angle of expectation violation and argues that perceived novelty of a new product decreases product evaluation because it violates evaluators’ expectations, increases the product’s ambiguities, and necessitates burdensome efforts to interpret the novelty [4, 22]. Without disputing these two perspectives, our model integrates their key theoretical arguments by finding an inverted U-shaped curve between an evaluator’s perceived novelty and his/her product evaluation. Although our integrative model is proposed in the context of new product evaluation, it is possible that this model is applicable to other evaluation contexts because we believe that these two contrasting perspectives our model integrates are common perspectives in human evaluation in general. For example, in presidential elections, citizens take the role of evaluators in assessing the election pledges of each candidate. Here, candidates may face a dilemma regarding how novel their election pledges should be—on the one hand, they may feel the need to generate very novel pledges to elicit curiosity and attract citizens’ attention; on the other hand, they may not want to provoke negative evaluations by making citizens feel violated by novel pledges. Applying our integrative model to the election context, we would expect to see that a moderate level of novelty has an advantage, which balances the amount of novelty and familiarity in candidates’ pledges. However, such generalizability requires empirical tests. Thus, future research testing our model in different evaluation contexts would be meaningful for both academic research and practitioners.

Second, our research shows the interesting irony of the role of a producer’s reputation. Generally, producers gain a reputation by demonstrating novelty in their new products. In the movie industry, for example, eminent awards, such as Academy Awards and BAFTAs, are given to directors who have developed movies with novel insights, contents, or technology [71]. In a sense, these awards motivate producers to create more novel products later in their careers. However, we found that a producer’s reputation gained by producing novel products in his/her past ironically penalized the producer’s subsequent novel products. This finding contributes to the literature on the reverse halo effect, whereby a person’s positive attributes hamper him/her in attaining desirable outcomes. Thus far, this effect has been investigated only in studies outside the literature on new product evaluation. For example, Sigall and Ostrove (1975) [72] found that physical attractiveness backfires when a focal person violates norms. More recently, M. Lee et al. (2018) [73] showed that the physical attractiveness of job candidates lowered their evaluations by recruiters if the job was socially less desirable. Our research contributes to the reverse halo effect literature by showing its generalizability to the context of new product evaluation.

Third, our research contributes to the novelty literature by providing empirical evidence regarding the effect of product novelty on evaluators’ perceived novelty of that product. Past studies that have examined product novelty have held an untested assumption that product novelty invariably predicts customers’ perceptions of the product’s novelty (see, for example [12, 44, 74]). Under this assumption, their theories have generally adopted the two-stage model relating product novelty to the success of the new product—i.e., product novelty affects evaluators’ perceived novelty of a product, which affects evaluators’ product evaluation and therefore ultimately affects the success of the new product (or idea). However, the two stepwise mechanisms were neither measured nor tested in their models, which might have contributed to the inconsistent empirical findings in the extant literature regarding product novelty and its success (see, for example [12, 44–46]). Our research offers an empirical examination of the relationship between product novelty and perceived novelty and finds that the relationship is nonsignificant. In other words, a product’s novelty failed to be perceived as novel by customers. We believe that our nonsignificant finding may explain why past research has produced inconsistent findings. Thus, future research that further investigates this inconsistency and the mechanism of the relationship between the producer’s side and the perceiver’s side of novelty would benefit the literature.

Finally, our additional analyses contribute to the customer engagement literature by providing another meaningful finding about how perceived novelty influences customer engagement, defined as “the intensity of an individual’s participation in and connection with an organization’s offerings and/or organizational activities, which either the customer or the organization initiate” [75]. An evaluator’s voluntary effort to review a new product is a type of customer engagement that significantly contributes to other customers’ use of the product [76]. Potential customers acknowledge that reviewing is an extra-role behavior that is not incentivized by producers, and thus, they are more receptive to effortful reviews [77]. We found that evaluators expended the most effort in reviews of new products with a moderate level of novelty. This finding implies a positive spiral of moderate novelty, which not only is the most beneficial to product evaluation but also facilitates more effortful reviews from evaluators that may attract future customers.

Our research also provides useful advice to managers in organizations. First, our study underlines that even though producers (e.g., companies, individual inventors) create novel products (i.e., product novelty), customers may perceive these products’ novelty in a completely different way. However, in evaluations of new products, perceived novelty may be more important than product novelty because customers base their purchasing decisions on their subjective perception of a new product [20, 78]. In fact, our findings showed that product novelty was unrelated to the final evaluation, while perceived novelty significantly influenced it. Thus, producers should pay greater attention to and constantly check their target customers’ perception of a product’s novelty and their product evaluation in the whole process of new product development (e.g., using focus groups). In this way, producers can align the projected novelty of their new products with customers’ actual perception of product novelty. Second, producers should maintain a balance between novelty and familiarity when developing their new products. Research has shown that producers, particularly creative producers, tend to be more interested in novelty than in familiarity and to constantly pursue greater novelty in their products [79, 80]. Our research shows that such a tendency of (creative) producers may backfire and make their products less desirable because evaluators appreciate only moderate levels of novelty in a new product. Thus, we suggest that producers (particularly creative producers) may need to refrain, at least to some degree, from expressing novelty in their products. This restraint may be particularly necessary for producers with a high reputation, as evaluators expect more familiarity or consistency between these producers’ new products and old products.

### Limitations and future directions

Although our research utilizes a rich dataset and offers new theoretical insights for the literature, it has some limitations. First, our sample was collected only from the movie industry in the U.S., which may limit the generalizability of our findings. We chose the movie industry to investigate our model because the movie industry has a large number of new products and a large volume of evaluations and reviews from customers. Other industries possess such characteristics as well. For example, the publishing industry releases new books and obtains customer reviews for those books, and an R&D team generates new ideas and receives evaluations from both internal and external stakeholders. Moreover, our research used big data (49,835 reviews of 147 movies) to achieve as much external validity as possible for our findings. However, we acknowledge that future research testing our model in different industries in other countries will help demonstrate its generalizability. We believe that our computer-aided content analysis with big data approach can be applied to examine perceived novelty in other settings, for example, the appraisals of business proposals and the reviews of new technological innovations in various industries.

Second, our investigation does not offer causal evidence for the relationship between perceived novelty and product evaluation, as our findings are based on an archival correlation study. It is possible that reverse causality may exist—e.g., an evaluator first determines a rating for the product and then justifies the rating using novelty. Nevertheless, we believe this possibility is relatively low because if evaluators had tried to justify the rating using novelty, they would have used the novelty justification in a linear way—e.g., when using novelty to justify their predetermined high rating, they would have used many novelty-related words, which would have led us to find a linear positive relationship between perceived novelty and product evaluation. That is, it is difficult to imagine that the evaluator deliberately used novelty justification in such a way that a moderate level of novelty was construed as the most desirable level. However, we acknowledge that future researchers testing the causal relationship between perceived novelty and product evaluation in a controlled laboratory study can significantly contribute to the refinement of our integrative model of new product evaluation.

Third, our research shows that evaluators’ assessments are influenced by product-unrelated information (i.e., a producer’s reputation). In an ideal evaluation of a new product, evaluators would focus only on the central characteristics of the new product, such as its novelty, and discard any irrelevant information, such as a producer’s reputation. However, our findings showed that a producer’s reputation can interact with core characteristics and affect product evaluation. Given this evidence, future research investigating additional product-irrelevant information (e.g., a producer’s physical attractiveness, gender, and race; an evaluator’s motivation, personality, and values) that may bias an evaluator’s rating would help draw a fuller picture of new product evaluation processes [11].

Finally, our research shows that the link between product novelty and perceived novelty is not significant, which challenges the assumption of past research that perceived novelty is one of the key mechanisms connecting product novelty and new product success (see, for example [44–46]). However, given that past research has found a significant, although inconsistent, relationship between product novelty and product success, there may be other mechanisms and contexts underlying this relationship. Therefore, future research that scrutinizes the mechanisms between product novelty and product success can significantly advance the novelty literature.

In closing, our integrative model of new product evaluation offers a comprehensive theoretical framework that incorporates two contradictory perspectives on how perceived novelty influences product evaluation. The integrative model shows that a moderate amount of perceived novelty is appreciated the most by evaluators. Furthermore, we found a reverse halo effect of a producer’s reputation such that evaluators expect less novelty for high reputation producers than from low reputation producers, which means that they penalized the novel products of high reputation producers more harshly. We hope that our work stimulates future researchers to apply our integrative model to diverse evaluation contexts in addition to the new product context.

## Supporting information

S1 File Data and code. (ZIP)
